# Proteomic variations after short-term heat shock treatment reveal differentially expressed proteins involved in early microspore embryogenesis in cabbage (*Brassica oleracea*)

**DOI:** 10.7717/peerj.8897

**Published:** 2020-04-08

**Authors:** Henan Su, Guo Chen, Limei Yang, Yangyong Zhang, Yong Wang, Zhiyuan Fang, Honghao Lv

**Affiliations:** 1 Hunan Agricultural University, Changsha, China; 2 Institute of Vegetables and Flowers, Chinese Academy of Agricultural Sciences, Beijing, China

**Keywords:** Cabbage, Microspore culture, Embryogenesis, Heat shock treatment, Proteomics

## Abstract

Microspore embryogenesis (ME), a widely used haploid breeding method that can considerably shorten the breeding cycle, provides an efficient mean of cultivating many important *Brassica* crops, such as cabbage, Chinese cabbage, and oilseed rape. For cabbage, in many cases, short-term heat shock treatment can strongly increase the embryogenesis rate, however, the underlying mechanism of this effect has not been elucidated. In this study, we compared the proteomics of isolated microspores with samples pretreated at 32 °C for 24 h and 25 °C for 24 h using two cabbage accessions (Zhonggan 628 and 87–534) showing highly different embryogenic rates. The embryo yield was 19.7 embryos/bud in Zhonggan 628 after 32 °C treatment, while no embryoid was observed in Zhonggan 628 after 25 °C treatment as well as in 87–534 at both temperatures. We identified a total of 363 and 282 differentially expressed proteins (DEPs) for Zhonggan 628 and 87–534 via a label-free proteomics technology. There were 97 DEPs specifically identified only in Zhonggan 628 but not in 87–534 after 32 °C heat-shock treatment that may be related to heat shock-induced embryogenesis in vitro culture. These DEPs were primarily enriched in carbon metabolic process, protein synthesis and degradation process, and signal transduction. Based on protein-protein interaction and pathway enrichment analyses, we proposed that SGT1 homolog A and B(SGT1), heat shock 70 kDa protein 5 (HSP70), cell division control protein 48 homolog A (CDC48) and fatty acyl-CoA reductase (FAR) might play important roles in microspore embryogenesis. This proteomic study may contribute to our molecular understanding of cabbage microspore embryogenesis and help to build a high-efficiency haploid breeding system.

## Introduction

Cabbage (*Brassica oleracea* L. var. *capitata* L.) is one of the most economically important cruciferous vegetables in the world, providing both nutrients and health promoting substances in human diets. The production of cabbage and other *Brassica* crops throughout the world was 71.26 million tons in 2016, with China producing nearly one third of them (http://faostat.fao.org/) (Ji et al., 2018). Most commercial cultivars are F_1_ hybrids that utilize heterosis, such as Zhonggan 21, Jingfeng 1, and Zhonggan 628 released in China (Lv et al., 2014). To produce the hybrids, we first need to cultivate high-generation inbred lines. In a traditional inbred line breeding method, the work is labor-intensive and normally requires 7–8 years; at the same time, microspore culture, a widely used method of haploid breeding, provides an effective technique to produce homozygous doubled haploid (DH) parental lines in only one or two years, which significantly accelerates the breeding process (Abercrombie, Farnham & Rushing, 2005; Ahmadi et al., 2014; Ferrie & Caswell, 2011; Maraschin et al., 2005). In addition, doubled haploid (DH) populations are ideal materials for genetic analysis, linkage map construction and gene mapping (Germana, 2011).

Microspore embryogenesis is a process in which microspores at the optimum period are isolated from the flower buds and induced in vitro to form haploids (Hosp et al., 2007; Ahmadi, Ahmadi & Silva, 2018). The chromosomes of the haploids can be doubled by colchicine treatment or subcluture, and the double haploids (DHs) are genetically homozygous and can be used as breeding lines directly. To date, more than hundreds of plant species have been reported and are especially widely used in *Brassica* crop breeding programs (Telmer, Newcomb & Simmonds, 1995; Shmykova, Shumilina & Suprunova, 2016; Shumilina, Shmykova & Bondareva, 2015). In recent years, many researchers have studied isolated microspore culture technology in *Brassica* and strongly improved the microspore embryogenesis rate (Yuan et al., 2011; Shumilina, Shmykova & Bondareva, 2015). In particular, extensive studies have shown that pretreatment, usually performed under heat or cold treatment, is essential for *Brassica* embryo induction (Joosen et al., 2007; Yuan et al., 2011; Yuan et al., 2012). In *B. napus,* embryogenesis could be induced after incubation at 32 °C or 18 °C for least 8 h (Pechan et al., 1991; Custers et al., 1994). In broccoli (*B. oleracea* L. var. *italica* L.), the optimum pretreatment was cold (4 °C) treatment for 24 or 48 h and then heat shock (32 °C) for 24 h (Yuan et al., 2011). However, in cabbage, microspore embryogenesis was efficiently induced after heat shock (32 °C) for 24 h (Yuan et al., 2012; Takahata & Keller, 1991). Although heat shock treatment plays an important role in cabbage during in vitro microspore embryogenesis, its underlying molecular mechanism has not been fully elucidated.

The omics method is now providing new opportunities for identifying the molecular mechanisms underlying plant growth and development, such as transcriptomics and proteomics (Xing et al., 2019; Sun et al., 2013). A number of studies have investigated the genes responsible for microspore embryogenesis in many important plant species, including rapeseed (Soriano, Li & Boutilier, 2013; Boutilier et al., 2002), tobacco (Zarsky et al., 1992; Touraev et al., 1996a) and wheat (Indrianto et al., 2001). These studies have identified some key genes whose expression is responsible for stress-induced microspore embryogenesis. However, examining mRNA in transcriptomics studies cannot fully represent the true level of protein expression, and the molecular changes under certain conditions can be more directly clarified by analyzing the protein profiles, as proteins are the executors of physiological functions (Kaspar-Schoenefeld et al., 2016; Domzalska et al., 2017). Therefore, proteomics technology has been widely used to explore the molecular mechanisms underlying a variety of plant growth and development (Balbuena, Jo & Pieruzzi, 2011; Balbuena et al., 2009; Rode et al., 2012).

Protein identification by two-dimensional gel electrophoresis (2-DE) and mass spectrum (MS) is a commonly used method in plant proteomics. To date, the combined use of 2-DE and MS has enabled us to identify a number of proteins related to somatic embryogenesis (Almeida et al., 2012; Correia et al., 2012; Sharifi et al., 2012). In comparison, only a limited number of studies have investigated proteome changes associated with microspore embryogenesis in *Brassica* (Pechan et al., 1991; Cordewener, Bergervoet & Liu, 2000; Joosen et al., 2007). Pechan et al. (1991) identified some heat-shock proteins induced by heat treatment of *B. napus* microspores and pollen. In addition, Joosen et al. (2007) identified 32 upregulated proteins associated with such processes as carbohydrate metabolism, redox reactions, and protein synthesis in *B. napus* during the development process of microspore-derived embryos. However, 2-DE offers a low resolution and cannot detect low-abundance proteins (Patel et al., 2009; Zhu, Smith & Huang, 2010). At the same time, the label-free quantification method, which evaluates the total number of allocated MS/MS spectra for peptides from a given protein, can greatly improve the detection efficiency (Lee & Koh, 2011). Therefore, it is desirable to identify a number of key proteins related to microspore embryogenesis by using a label-free quantification method.

Currently, microspore culture is widely applied in cabbage breeding, while short-term heat shock treatment is important for embryogenesis. However, the underlying molecular mechanism governing this phenomenon remains largely uncharacterized. In this work, we identified differentially expressed proteins after 32 °C and 25 °C treatments for 24 h in isolated cultured microspores of cabbage accessions Zhonggan 628 (high embryo yield of 19.7 embryos/bud) and 87–534 (low embryo yield of 0 embryo/bud) by using a label-free proteomic approach. Our study focused on the development process between the non-embryo and embryo microspores, identifying proteins that are differentially expressed. The information obtained from protein profile analysis will broaden our understanding of the molecular regulation of microspore embryogenesis induced by short-term heat shock treatment.

## Material and Methods

### Plant materials, treatment, and sample collection

Cabbage accessions Zhonggan 628 (excellent in the embryo yield of microspore culture) and 87–534 (exhibiting a low yield in microspore-derived embryos) were used in this study. They were cultivated in a greenhouse (20−25 °C with a 16 h/day photoperiod of natural light) of the Institute of Vegetables and Flowers, Chinese Academy of Agricultural Sciences, Beijing, China. The microspore isolation and culture process was carried out from mid-March to early May in their flowering stage, as previously described by Lv et al. (2014). Briefly, seventy-eight buds for each accession with a length of 3–3.5 mm were selected for cultivation after sterilization and microspore isolation. According to our previous study, this length represented the premium sampling stage that provided the highest embryo rate for these cabbage accessions, with 60%–70% of the micropores being at the mid- to late-uninucleate stage (Su et al., 2018). NLN-13 was used as the liquid microspore culture medium, and the microspores was adjusted to a density of 1 ×10^5^/ml in the liquid medium containing 1 ml suspension and 1 ml NLN-13 in each petri dish. Then, they were induced at 32 °C and 25 °C (as an untreated control) for 24 h, respectively, and finally incubated at 25 °C in the dark. Three weeks later, the microspore embryogenesis rates were counted. Six petri dishes of each sample were randomly chosen to determine embryogenesis statistics, with three replicates. For molecular analysis, after 32 °C and 25 °C treatments for 24 h, twenty petri dishes of the isolated microspores for Zhonggan 628 and 87–534 were collected by centrifugation (5 min at 100× g), respectively, with three replicates. These collected samples were immediately frozen in liquid nitrogen and stored at −80 °C for subsequent extraction of protein and total RNA.

### Plant sample collection and protein extraction

The collected microspore samples were ground into powder under liquid nitrogen and then dissolved in 1 ml of extraction solution (150 mM Tris-HCl (pH 7.6), 8 M urea, 0.5% SDS, 1.2% Triton X-100, 20 mM EDTA, 20 mM EGTA, 50 mM NaF, 1% glycerol, and 5 mM DTT, PI). The samples were centrifuged at 10,000× g for 1 h at 4 °C to collect the supernatants, which were then mixed with precooled acetone/methanol solution for three times the supernatants’s volume and incubated for 1 h at −20 °C. The mixed samples were centrifuged at 15,000× g for 15 min at 4 °C, and the precipitated proteins were rinsed 1–2 times with precooled acetone. Subsequently, the supernatants were discarded after 15,000× g for 5 min at 4 °C. The precipitated proteins were naturally air-dried at room temperature and redissolved in 100 µL of lysis buffer containing 50 mM Tris-HCl (pH 6.8), 8 M urea, 5 mM DTT, 1% SDS, and 10 mM EDTA. The protein precipitation and resuspension cycles were repeated twice. The resulting total proteins were measured using the Bradford assay method (Bradford, 1976). Then, 60 µg of the protein solution were transferred to a centrifuge tube, mixed with 5 µl of 1 M DTT solution, and incubated at 37 °C for 1 h. Then, 20 µl of 1 M IAM solution was added, mixed and reacted at room temperature for 1 h in the dark. After that, all samples were pipetted into an ultrafiltration tube; the collection solution was discarded after centrifugation, and 100 µl UA (8 M urea, 100 mM Tris-HCl, pH 8.0) was added to the tube; this step was repeated twice. Then, 100 µl of 50 mM NH_4_HCO_3_ was added, and the collection solution was discarded after centrifugation; this step was repeated for three times. Finally, the collected samples were digested with trypsin, with the ratio of 50:1 (protein vs. enzyme) in an ultrafiltration tube at 37 °C for 16 h. The digested samples were stored at −80 °C until MS analysis (Zhu et al., 2013).

### Liquid chromatography-tandem mass spectrometry/mass spectrometry (LC-MS/MS) analysis

The digested peptide mixtures were transferred to a fused silica capillary column packed with 3-µm Dionex C18 material (Dionex, Sunnyvale, USA). The 15-cm RP column sections were washed with buffer A (H_2_O and 0.1% formic acid) and buffer B (acetonitrile and 0.08% formic acid). A C18 capture tip (five mm, 0.3 mm) was laid up in an Agilent 1100 quaternary high-performance liquid chromatography (HPLC) (Agilent Technology Co. Ltd., Santa Clara, CA, USA) in a straight line after desalting; subsequently, a 12-step separation method was used for analysis (Pang et al., 2016). Gradient elution was carried out as follows: buffer B started at 2% to 40% in 45 min, followed by a 13-min gradient to 80% buffer B; next, the buffer B flowed from 80% to 2% until 2 min. The separated peptides were examined in a micrOTOF-Q II mass spectrometer (Bruker Corporation, Billerica, MA, USA) with a source temperature of 180 °C. The mass spectrometer was run in automatic mode: survey MS scans were performed with a resolution setting of 20,000 followed closely behind by five data-dependent tandem mass (MS / MS) scans at 2 Hz normalized scan speed (Pang et al., 2016).

### Data processing for proteomics

The MS data were searched against the *B. oleracea* genome (http://plants.ensembl.org/index.html) and were processed using Proteome Discover software (Version 1.3, Thermo Scientific, Waltham, USA), with the parameters as follows: carbamidomethylation (C), oxidation (M), and acetylation (Protein N-term) were set to immutable and variable compositions, respectively; 15 ppm and 20 mmu were peptide mass tolerance and fragment mass tolerance, respectively; max missed cleavages was 2. A cutoff of 1% for peptide false discovery rate (FDR) was used for peptide and protein identification, and peptides with Z score <4 or Delta-Mass >5 ppm were discarded. In addition, proteins with at least one unique peptide were characterized. These proteins with fold-change ≥ 1.5 and *p*-value (*p-* FDR) ≤ 0.05 was deemed as differentially expressed proteins (DEPs) between the experimental groups (Ji et al., 2018).

### Bioinformatic analysis

A bioinformatic analysis was conducted to categorize the proteins based on biological processes, cellular competence and molecular function by using the Evolutionary Relationships (PANTHER) database v6.1 (http://www.pantherdb.org), following the gene ontology (GO) standards (Thomas et al., 2003). Moreover, pathway analyses were carried out on the Kyoto Encyclopedia of Genes and Genome (KEGG) database (http://www.Genome.jp/kegg) for biological interpretation of systemic functions (Cho & Campbell, 2000). Protein-protein interactions were analyzed on the Retrieval of Interacting Genes/Proteins (STRING) database (http://string-db.org) including known and predicted protein-protein systemic functions (Jensen et al., 2009).

### Total RNA extraction and qRT-PCR analysis

The transcript levels of genes involved in DEPs were identified using quantitative real-time- polymerase chain reaction (qRT-PCR). Total RNA was isolated separately from Zhonggan 628 isolated microspores under 32 °C and 25 °C treatments for 24 h, using a TaKaRa MiniBEST Plant RNA Extraction Kit (TaKaRa, Dalian, Liaoning, China) following the manufacturer’s protocol (https://www.takarabiomed.com.cn). A RevertAid First Strand cDNA Synthesis Kit (TaKaRa) was used to reversely transcribe RNA to cDNA. The qRT-PCR reactions were conducted as previously described (Su et al., 2019). *Actin* was used as the internal reference gene in cabbage. The relative expression levels of the target genes (*Bo5g021500*, *Bo8g066630*, *Bo2g023100*, *Bo6g031300*, *Bo3g045210*, *Bo2g165560*, *Bo1g050780*, *Bo5g138000*, and *Bo5g139630*) were calculated by using the 2^−^^△△^^Ct^ method (Schmittgen & Livak, 2008), and the test was repeated three times. The cDNA was amplified using specific primers (Table S1).

### Data analysis

Data were analyzed using SPSS Statistics 17.0 (IBM Corp., Armonk, NewYork, USA). One-way ANOVA and Tukey test were performed at 95% confidence level (*p* < 0.05) to assess whether the data of the treatments were significantly different.

## Results

### Microspore collection and culture

**Figure 1:**
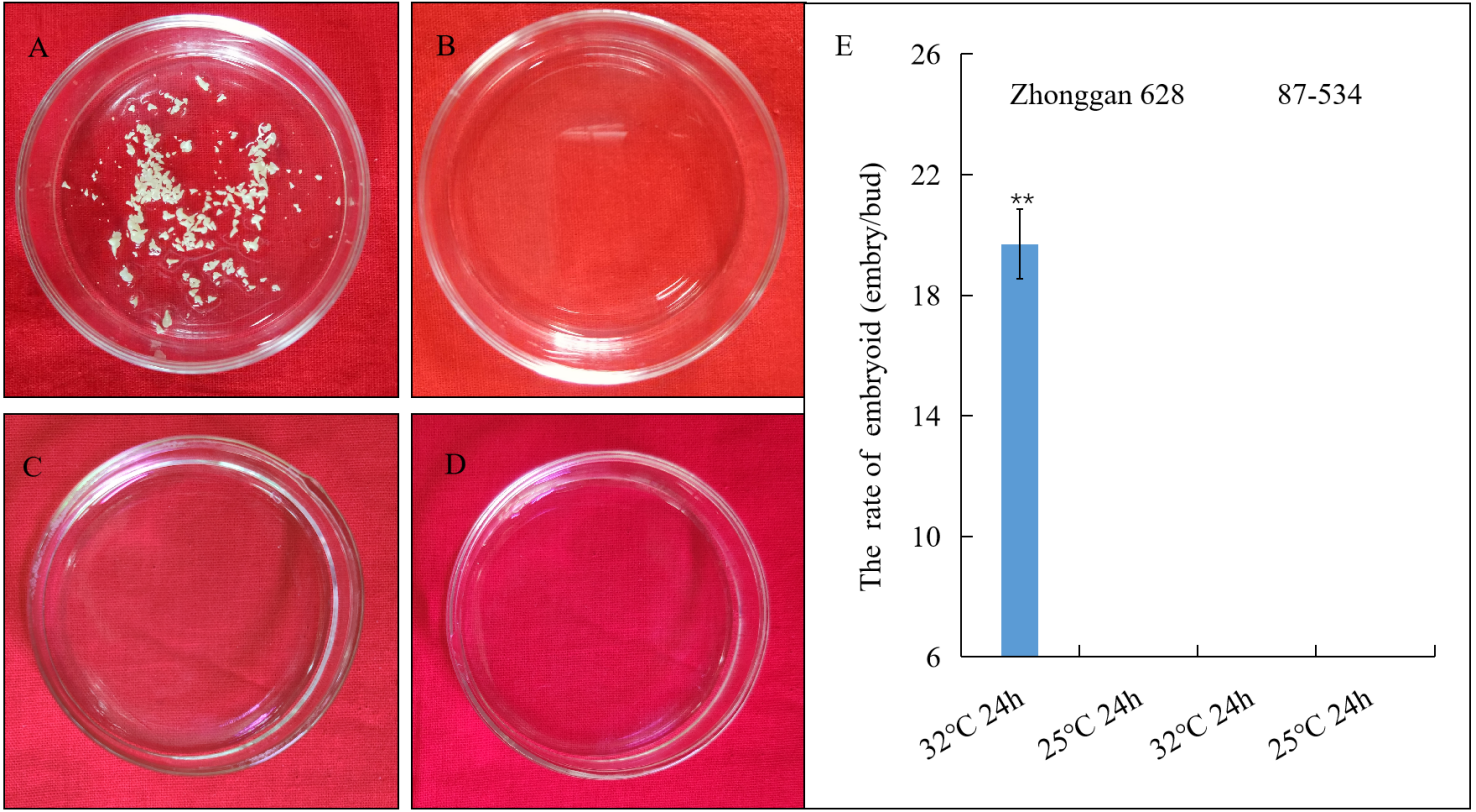
Embryoid observation and statistical analysis of embryoid rate under heat shock treatment of Zhonggan 628 and 87–534 microspores in vitro. (A) The embryoids of Zhonggan 628 after 32 °C treatment for 24 h. (B) The embryoids of Zhonggan 628 under 25 °C for 24 h. (C) The embryoids of 87–534 after 32 °C treatment for 24 h. (D) The embryoids of 87–534 under 25 °C for 24 h. (E) The rate of embryoid for Zhonggan 628 and 87–534 under 32 °C and 25 °C treatment for 24 h. (** *P* < 0.01).

The results showed that after three weeks, 19.7 embryos/bud were produced from the isolated microspores in Zhonggan 628 after 32 °C treatment (Fig. 1A), while no embryoid was observed in Zhonggan 628 after 25 °C treatment (Fig. 1B) as well as in 87–534 after both 32 °C and 25 °C treatment (Figs. 1C, 1D), and the difference in the embryo rates between the 32 °C and 25 °C treatments for 24 h in Zhonggan 628 was significant (** *p* < 0.01) (Fig. 1E). This result was similar to previous studies showing that 32 °C heat treatment could significantly improve the embryogenesis rate of cabbage (Yuan et al., 2012; Takahata & Keller, 1991).

### Identification of differentially expressed proteins in embryogenesis caused by high temperature

**Figure 2:**
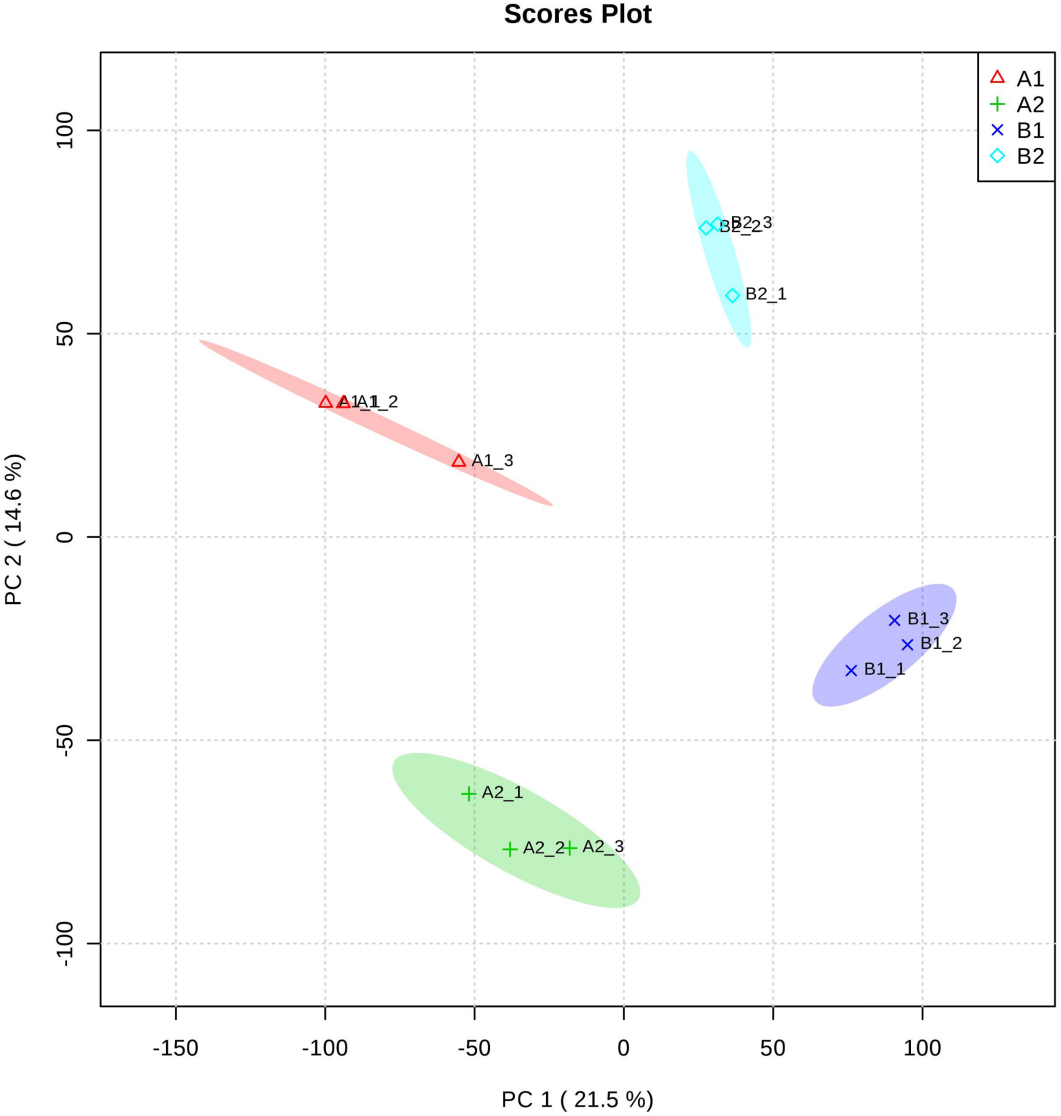
Principal component analysis at the proteome level. (A1) Zhonggan 628 under 25 °C for 24 h. (A2) Zhonggan 628 under 32 °C for 24 h. (B1) 87–534 under 25 °C for 24 h. (B2) 87–534 under 32 °C for 24 h.

**Figure 3:**
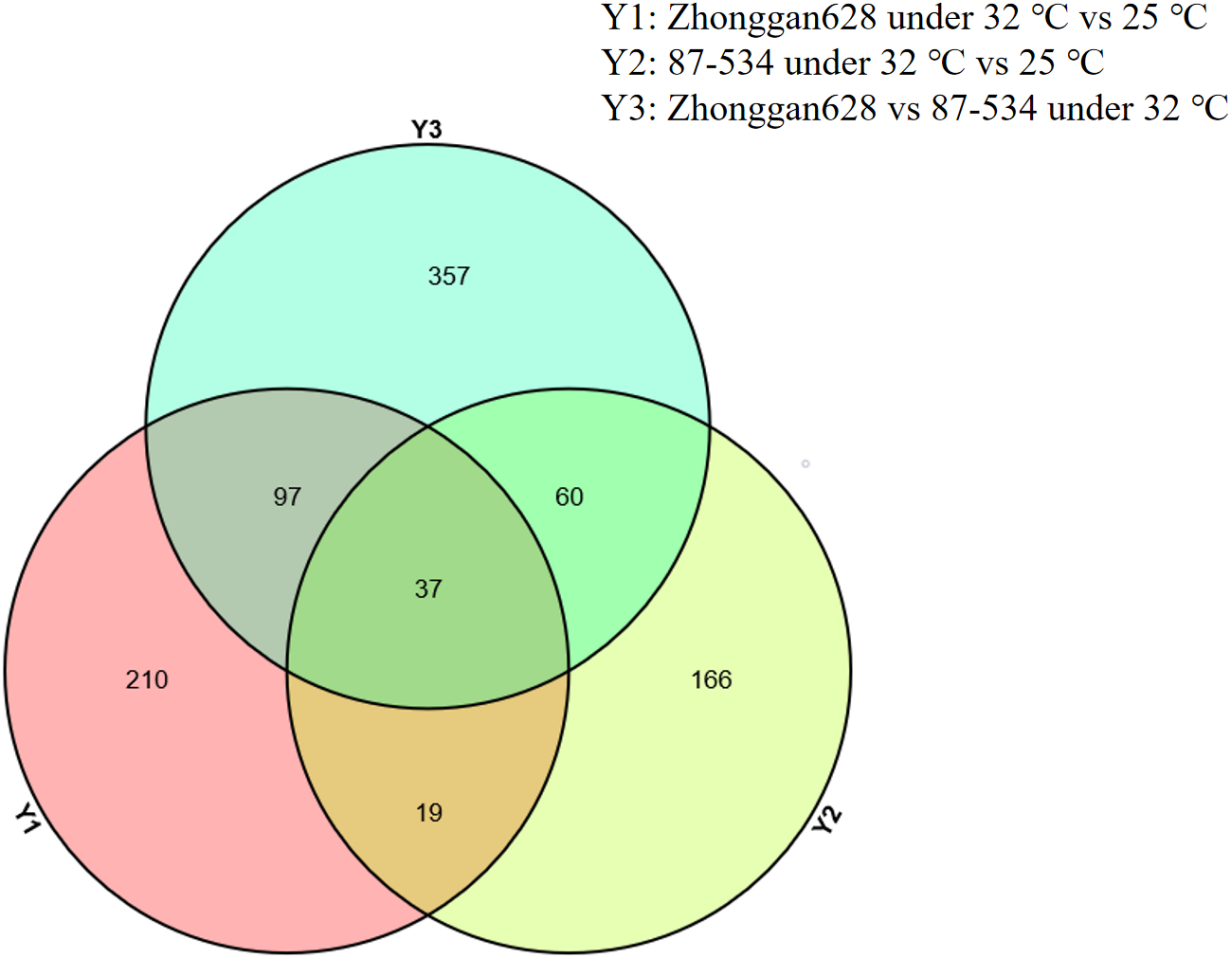
Venn diagrams showing numbers of distinct and common proteins in each of the three groups. (Y1) Zhonggan 628 under 32 °C vs. 25 °C for 24 h. (Y2) 87–534 under 32 °C vs. 25 °C for 24 h. (Y3) Zhonggan 628 under 32 °C for 24 h vs. 87–534 under 32 °C for 24 h.

To decipher the proteomic variations after treatments at 32 °C and 25 °C for 24 h in cultured microspores, highly embryogenic cultivar Zhonggan 628 (A) and 87–534 (low yield in microspore-derived embryos, B) after treatments at 32 °C and 25 °C (as untreated control), respectively, for 24 h were chosen for the proteomic comparisons. Using label-free analysis, we identified a total of 33434 peptides and 5961 protein species among the 12 samples. A clear separation between A and B was found from the principal component analysis (Fig. 2). Proteins with more than 1.5-fold changes in abundance (*p* ≤ 0.05) between the HS treatment (32 °C) and CK (25 °C) were selected as differentially expressed (Ji et al., 2018). We identified a total of 363 proteins showing expression differences in mass spectrometry from Zhonggan 628 (A) between 32 °C and 25 °C; among these proteins, 115 were downregulated, and 248 were upregulated (Table S2). In 87–534, a total of 282 proteins showing expression differences in mass spectrometry were identified between 32 °C and 25 °C; among these proteins, 162 were downregulated, and 120 were upregulated (Table S2). The results indicated that Y1 (Zhonggan 628 under 32 °C vs. 25 °C for 24 h) and Y3 (Zhonggan 628 under 32 °C for 24 h vs. 87–534 under 32 °C for 24 h) had 134 DEPs in common; among those 134 DEPs, there were 37 DEPs in Y2 (87–534 under 32 °C vs. 25 °C for 24 h) (Fig. 3); therefore, there were only 97 DEPs specifically identified only in Zhonggan 628 but not in 87–534 after 32 °C heat-shock treatment (Table S2), which might be the key proteins that warrant further investigation.

### GO function classification of differentially expressed proteins

**Figure 4:**
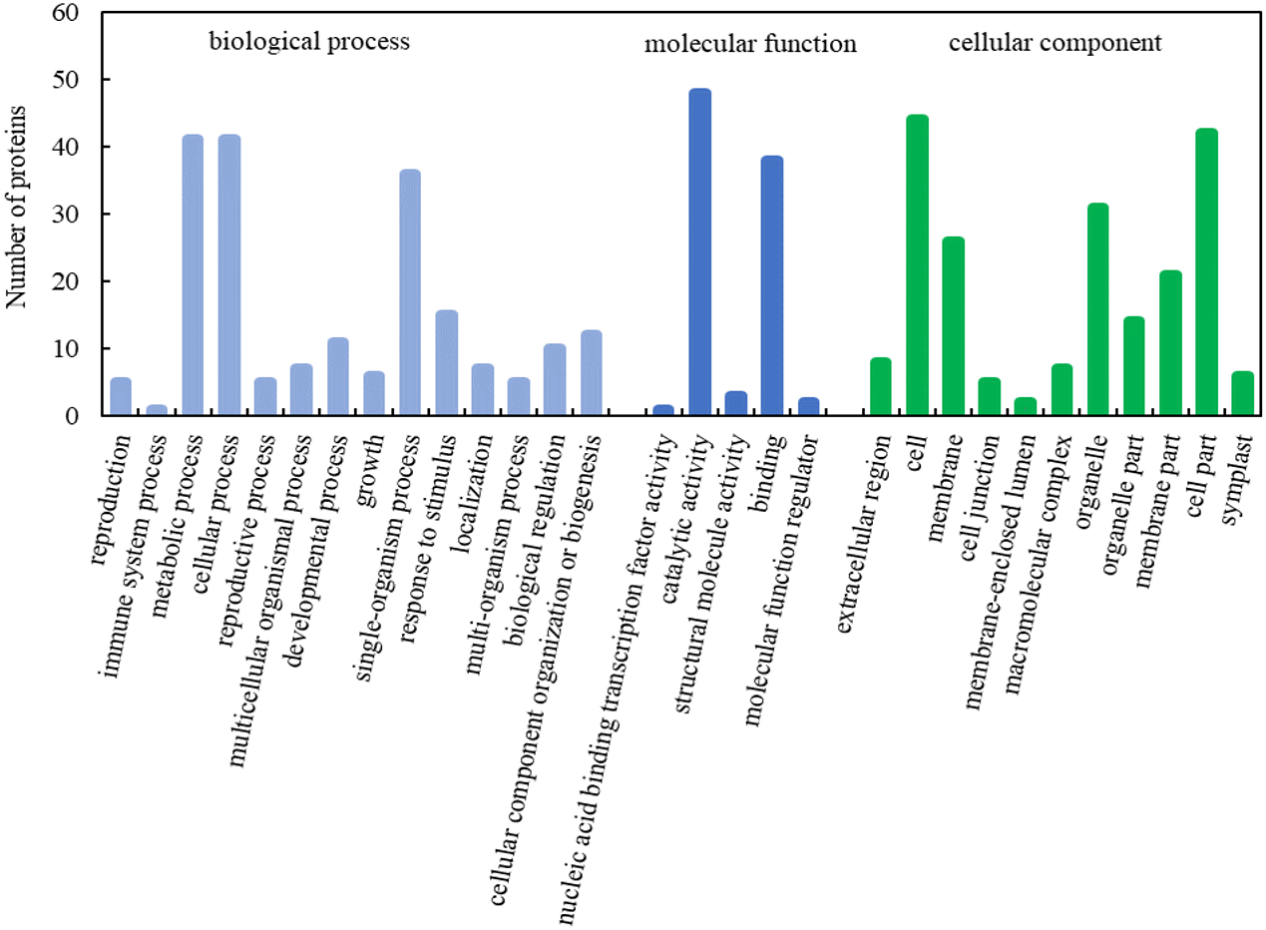
GO function classification of DEPs during embryogenesis caused by high temperature.

To further understand the function of the differentially expressed proteins, 97 key DEPs were searched in the database to obtain their functional information. The DEPs could be divided into three categories: cellular component, biological process, and molecular function (Fig. 4, Table S3). Based on cellular component, 97 key DEPs were classified into eleven categories, and the largest functional category included proteins involved in cell (GO:0005623), followed by those participating in cell parts (GO:0044464). Based on biological process, 97 key DEPs were classified into fourteen categories, and the largest functional category included proteins involved in metabolic process (GO:0008152). Based on putative molecular functions, the identified proteins were classified into five categories, and the largest functional category included proteins involved in catalytic activity (GO:0003824).

### KEGG pathway analysis of differentially expressed proteins

**Figure 5:**
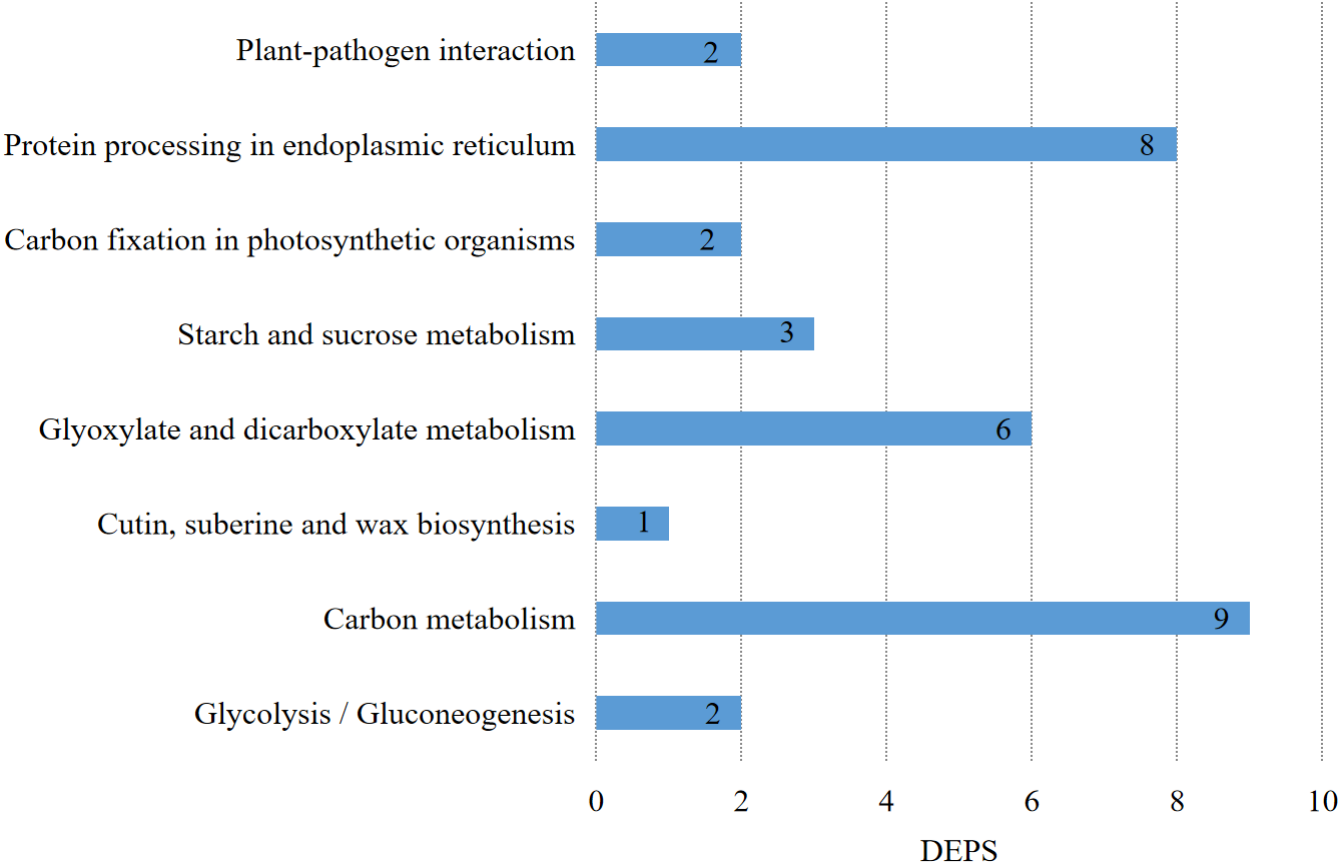
KEGG pathway of DEPs during embryogenesis caused by high temperature.

Metabolic pathways corresponding to the proteins could be found in the KEGG ORTHOLOGY (KO) (https://www.genome.jp/kegg/ko.html) database according to their KO numbers. 24 proteins had KO numbers among the 97 key DEPs, and they were enriched in carbon metabolism, glyoxylate and dicarboxylate metabolism, starch and sucrose metabolism, protein processing in the endoplasmic reticulum, glycolysis/gluconeogenesis, carbon fixation in photosynthetic organisms, plant-pathogen interaction, and cutin, suberine and wax biosynthesis. The results of the KEGG pathway analysis of the DEPs after HS-responsive embryogenesis (Fig. 5, Table S3) showed that carbon metabolism included nine DEPs, protein processing in the endoplasmic reticulum included eight DEPs, plant-pathogen interaction included two DEPs, and cutin, suberine and wax biosynthesis included one DEP.

The pathways of the DEPs were primarily involved in protein process and carbon metabolism, while some proteins were also enriched in a different pathway. There were a number of proteins involved in multiple pathways; for example, ribulose bisphosphate carboxylase small chain (RBCS) was involved in three different pathways (glyoxylate and dicarboxylate metabolism, carbon metabolism, and carbon fixation in photosynthetic organisms), while sugar isomerase family protein (SIS) and glucose-6-phosphate isomerase (GPI) also participated in more than two pathways.

### Interaction of differentially expressed proteins in embryogenesis caused by high temperature

**Figure 6:**
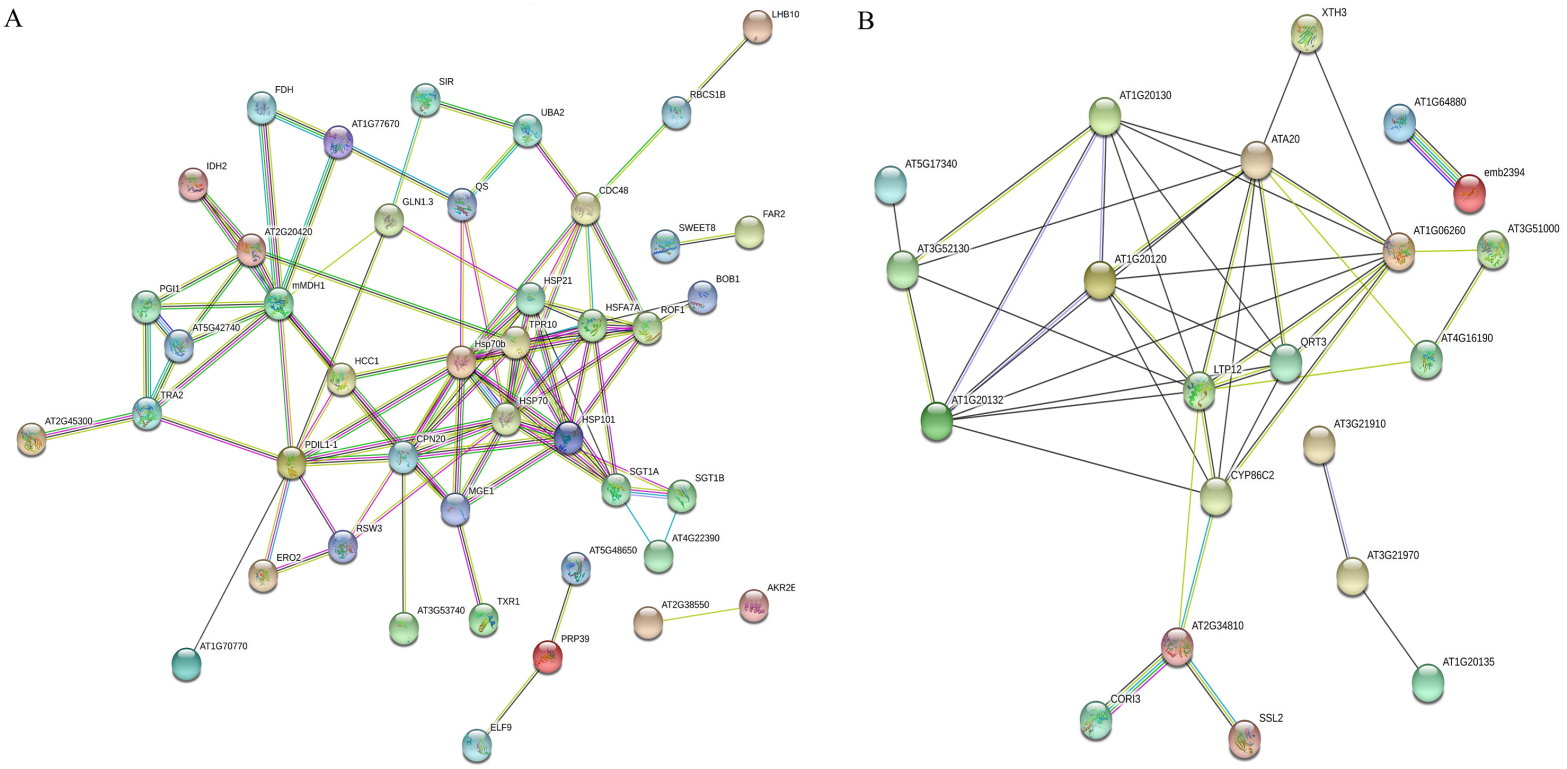
Protein–protein interaction analysis between DEPs. (A) The upregulated proteins. (B) The downregulated proteins.

We used the STRING database to construct the interaction networks of the 97 DEPs (64 upregulated proteins and 33 downregulated ones) by using an *Arabidopsis* association model. The results showed that 64 upregulated and 33 downregulated proteins were interacted with 32 and 14 target proteins, respectively (Fig. 6, Table S4). As expected, the network showed general and complex interactions among the 97 proteins. For example, among those 64 upregulated proteins, hsp21 description (HSP21, corresponding to Bo1g050780.1), putative ankyrin repeat protein RF (TPR10, corresponding to Bo1g151900.1), heat stress transcription factor B-4b-like (HSFA7A, corresponding to Bo2g165560.1), SGT1 homolog B (SGT1, corresponding to Bo3g045210.1), chaperone protein ClpB1 (HSP101, corresponding to Bo6g118620.1), and cell division control protein 48 homolog A (CDC48, corresponding to Bo5g138000.1) were closely interacted with heat shock 70 kDa protein 5 (HSP70, corresponding to Bo5g021500.1, Bo8g066630.1). In addition, 20 kDa chaperonin (CPN20, corresponding to Bo2g023100.1), malate dehydrogenase (mMDH, corresponding to Bo6g031300.1), and protein SCO1 homolog 1 (HCC, corresponding to Bo5g139630.1) showed highly positive interactions with protein disulfide-isomerase (PDIL1-1, corresponding to Bo8g071060.1). For those 33 downregulated proteins, cytochrome P450, family 86, subfamily C (CYP86C2, corresponding to Bo9g076040.1), nonspecific lipid-transfer protein (LTP12, corresponding to Bo1g059070.1), polygalacturonase QRT3 (QRT3, corresponding to Bo1g022160.1), and AT1G06260 (corresponding to Bo1g055000.1) were closely interacted with AT1G20120 (corresponding to Bo5g029180.1, Bo7g061770.1, and Bo7g061790.1). Although these estimated interaction networks require verification, they may provide a brief protein-protein interaction model in cabbage embryogenesis induced by high temperature, which might deserve further investigations.

### Correlation of protein fold changes with transcripts

**Figure 7:**
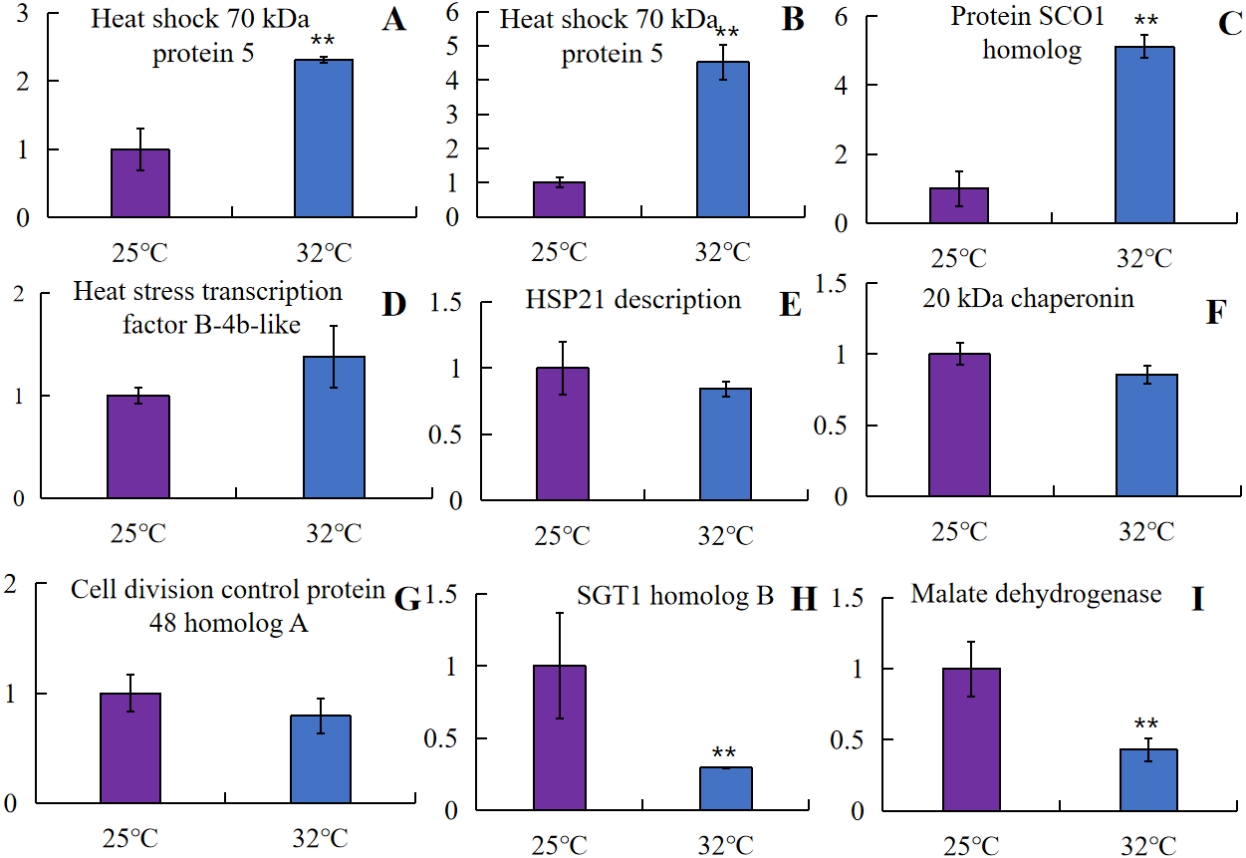
mRNA expression level analysis using a qRT-PCR approach on candidate proteins of embryogenesis caused by high temperature. (A) Bo5g021500. (B) Bo8g066630. (C) Bo5g139630. (D) Bo2g165560. (E) Bo1g050780. (F) Bo2g023100. (G) Bo5g138000. (H) Bo3g045210. (I) Bo6g031300 (** *P* < 0.01).

The protein and mRNA expression levels were correlated to further examine the relative abundance of proteins involved in embryogenesis caused by high-temperature treatment. The mRNA expression levels were acquired by using qRT-PCR analysis of the corresponding nine genes with more nodes in the interaction network. In the selected proteins, four genes showed similar change trends as the result of label-free proteomics. As was shown in Fig. 7, heat shock 70 kDa protein 5 (Bo5g021500, Bo8g066630), one of the most abundant proteins isolated from the microspore at 32 °C, was chosen for test, and as expected, a significant increase in two *HSP70* genes and one gene encoding SCO1 homolog 1 (HCC) expression was observed at high temperatures (** *p* < 0.01). One gene encoding heat stress transcription factor B-4b-like (HSF) showed a relatively increased abundance in Zhonggan 628 under high temperature. However, a significant decrease in two genes encoding mMDH and SGT1 expression was observed at high temperatures (** *p* < 0.01), the genes encoding proteins HSP21, CPN20, and CDC48 showed a relatively decreased abundance in Zhonggan 628 under high temperature, which displayed differences between label-free proteomics and qRT-PCR. This finding could be due to the transcription level changes from the genes to the proteins, such as translation regulation and posttranslational modifications.

## Discussion

In this work, we performed a comparative label-free proteomic analysis to analyze the dynamic protein profiles concerning microspore embryogenesis. Our results showed that the DEPs related to embryogenetic ability induced by high temperature were primarily enriched in carbohydrate metabolism, protein synthesis and degradation in the endoplasmic reticulum, signal transduction, and cutin, suberine and wax biosynthesis.

### Carbohydrate metabolic process

**Figure 8:**
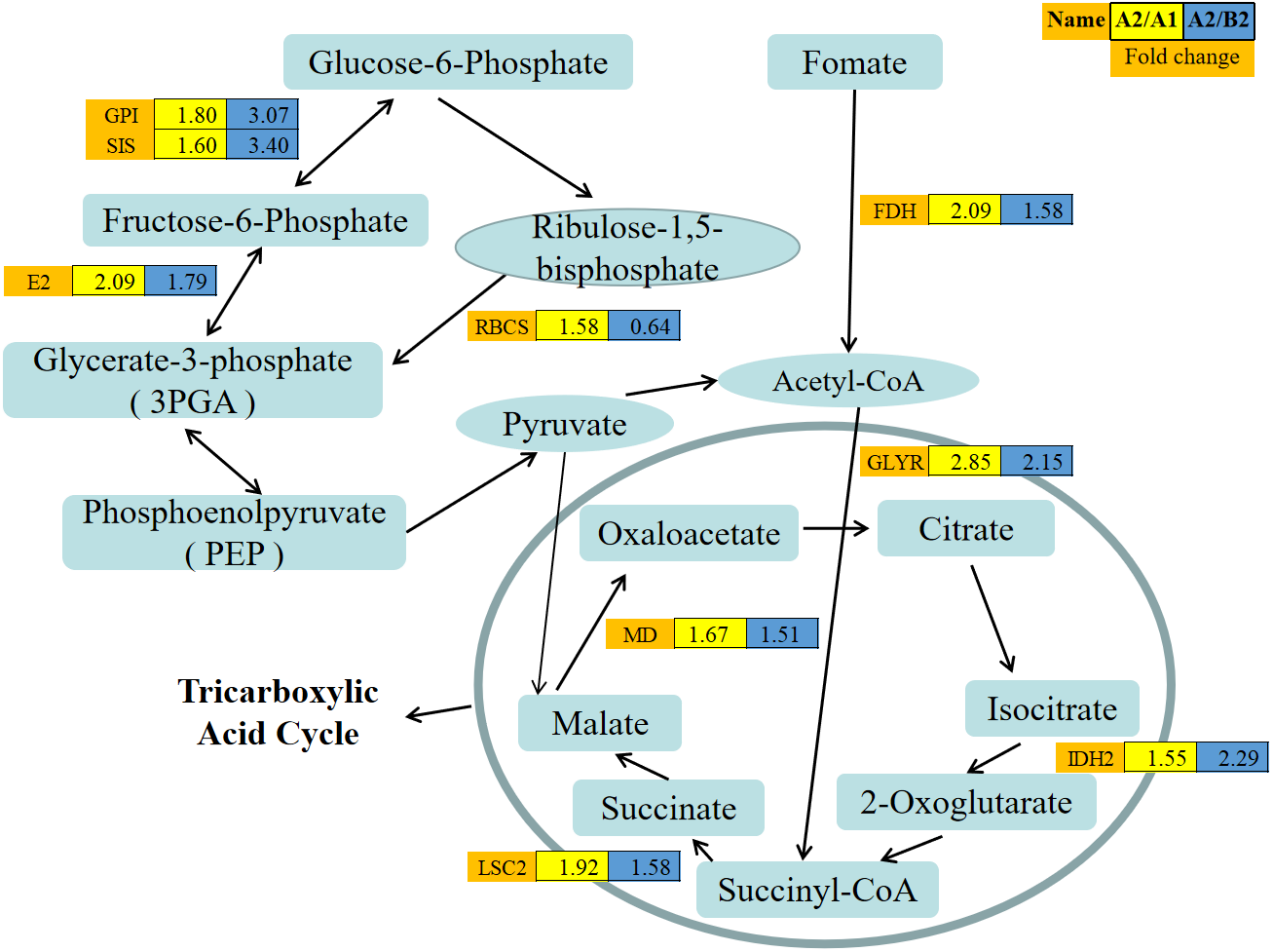
Abundance patterns of protein species involved in the carbohydrate metabolism. The expression value was log10 transformed. Yellow box (A2/A1): The expression levels of proteins from Zhonggan 628 under 32 °C vs. 25 °C; Blue box (A2/B2): The expression levels of proteins from Zhonggan 628 vs. 87–534 under 32 °C.

The induction of cabbage embryogenesis by high temperature may be associated with the carbohydrate metabolic process. In this study, a total of nine proteins were involved in carbohydrate metabolic process. Among the nine proteins, seven were related to the pentose phosphate pathway (PPP) and tricarboxylic acid cycle (TCA), and two participated in glyoxylate and dicarboxylate metabolism (Fig. 8, Table S5). Previous studies have indicated that carbohydrate metabolism is associated with the interruption of male gamete development (Mascarenhas, 1990; Maraschin et al., 2005). The comparative analysis between non-embryogenic and embryogenic callus indicated that most DEPs were involved in carbohydrate metabolism in maize (Varhaníková et al., 2014) and sugarcane (Heringer et al., 2015). In addition, the genes involved in sucrose-starch metabolism have also been described in barley microspore embryogenesis (Maraschin et al., 2006). Furthermore, the suppressed genes participating in starch biosynthesis have been reported to prevent pollen development in vivo (Datta, Chamusco & Chourey, 2002).

Glucose-6-phosphate isomerase (GPI),sugar isomerase (SIS) family protein, and transaldolase-like protein (E2) (Fig. 8) participated in the PPP and provided both nicotinamide adenine dinucleotide phosphate (NADPH) and pentose. Moreover, malate dehydrogenase (MD), isocitrate dehydrogenase subunit 2 (IDH), and succinate-CoA ligase [ADP-forming] subunit beta, mitochondrial LSC2 (LSC2) were linked to the tricarboxylic acid cycle (TCA), which were all upregulated in the 32 °C microspores. In addition to the cytoplasmic GPI, the chloroplasts of land plants harbor a nuclear-encoded isoenzyme of cyanobacteria for plastid starch accumulation (Grauvogel, Brinkmann & Petersen, 2007). MD can catalyze the reversible conversion between malate and oxaloacetate (Taylor et al., 1990). The high energy requirements were associated with the rearrangement of metabolism, cellular organization and regulation, and cell divisions, which explained why proteins related to carbohydrate metabolic process were most strongly expressed during early embryogenesis in vitro. This finding suggested that those upregulated proteins during heat shock treatment were activated to provide the energy needed for early microspore embryogenesis.

### Protein synthesis and degradation in the endoplasmic reticulum

**Figure 9:**
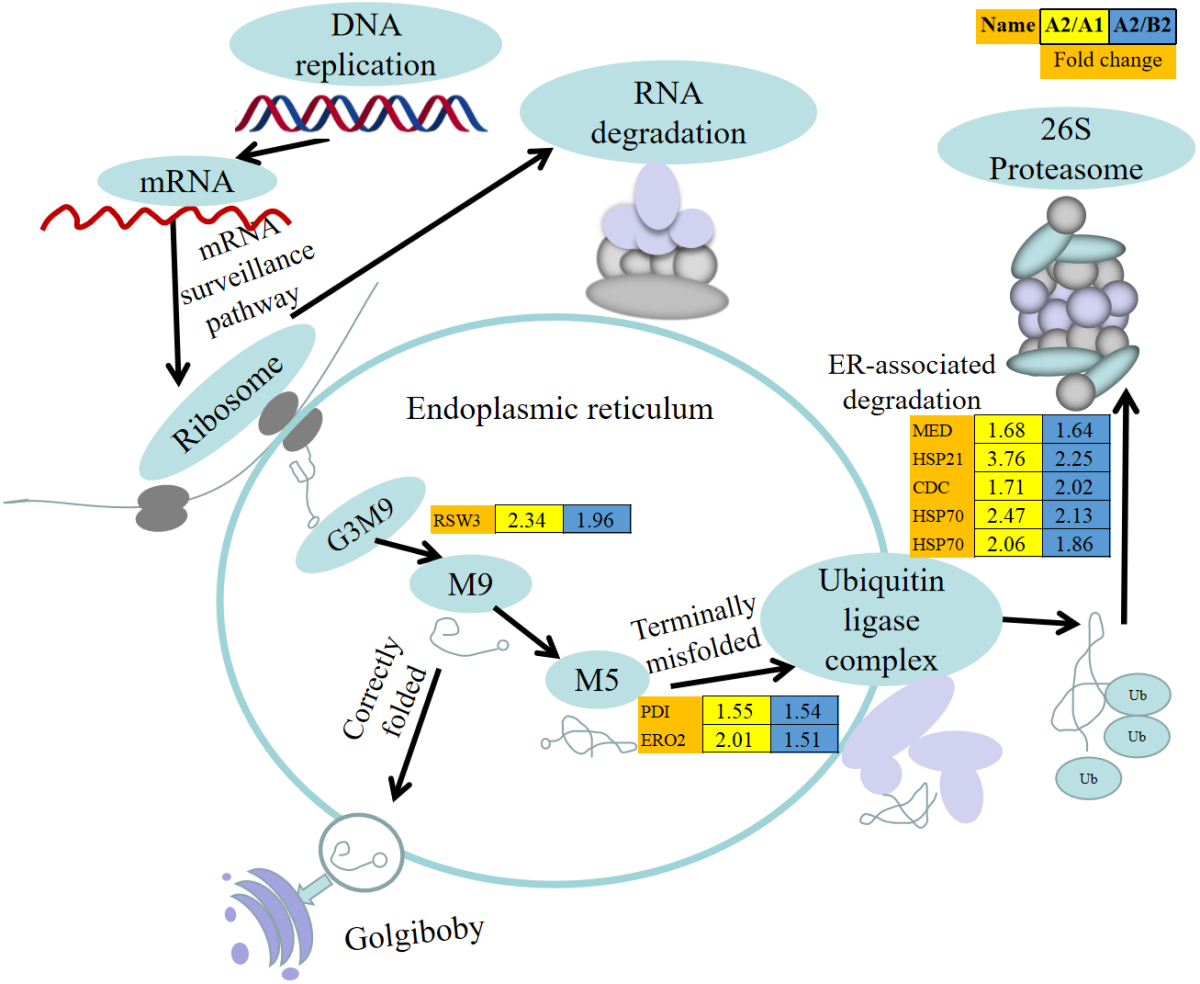
Abundance patterns of protein species involved in protein synthesis and degradation. The expression value was log10 transformed. Yellow box (A2/A1): The expression levels of proteins from Zhonggan 628 32 °C vs. 25 °C; Blue box (A2/B2): The expression levels of proteins from Zhonggan 628 vs. 87–534 under 32 °C.

Protein synthesis and degradation are the basis of life and the link between genetic information storage in DNA and protein. In this study, we discovered eight DEPs in protein synthesis and degradation process in the endoplasmic reticulum (Fig. 9, Table S5). The endoplasmic reticulum plays a key role in the protein folding of plant cells, while this process is vulnerable to environmental stresses. Moreover, eight ER-associated proteins including glycosyl hydrolase family 31 protein (RSW3), protein disulfide-isomerase (PDI), ERO2 description (ERO2), probable mediator of RNA polymerase II transcription subunit 37c (MED), HSP21 description (HSP21), HSP70, and cell division control protein 48 homolog A (CDC48) were identified in our study (Fig. 9).

The oxidation and isomerization of disulfide bonds are necessary for the growth of all organisms, and PDI and ERO1 form disulfide bonds, all of which undergo protein oxidation with redox reactions (Yudina, 2012). In maize, the primary PDI accumulated in seeds that produced mutant storage proteins and triggered the induction of the ER stress response (Geng et al., 2018). In barley, proteins involved in the ubiquitin-26S proteasomal pathway are induced in stressed enlarged barley microspores (Maraschin et al., 2006). In this work, CDC48 was involved in the ubiquitin-26S proteasomal pathway, and the expression ratios of CDC48 in Zhonggan 628 under 32 °C vs. 25 °C and Zhonggan 628 under 32 °C vs. 87–534 under 32 °C were 1.71 and 2.02, respectively. HSP70 in the cytosolic HSP family accumulates rapidly to regulate plant growth and development under various environmental stresses (Park & Seo, 2015). In addition, during transient stress, the expression of sHSPs (HSP18, HSP21, HSP22, and HSP23) was constantly increased by stress, and after upregulation they may become the most abundant cellular proteins (Wang et al., 2004; Haslbeck et al., 2005; Segu-Simarro, Testillano & Risuenn, 2003). Heat shock proteins (HSPs) are known to protect proteins by assisting in refolding, preventing aggregation or by acting as a cochaperone (Jan et al., 1995; Bélanger et al., 2018). Due to the chaperone activity, HSPs may play an indirect role in triggering androgenesis by controlling the subcellular localization of other key regulatory proteins or providing higher levels of heat resistance (Maraschin et al., 2005). In previous studies, members of the heat shock protein family have been reported to be highly expressed during the induction of microspore embryogenesis by heatshock and starvation treatment: HSP90 and HSP70 in *Brassica* (Cordewener, Bergervoet & Liu, 2000; Segu-Simarro, Testillano & Risuenn, 2003), as well as HSP20 in tobacco (Zarsky et al., 1995). In this study, the HSPs, such as HSP70 and HSP21 (Fig. 9), were all increasingly accumulated in 32 °C microspores, indicating their probable roles in stress induction of microspore embryogenesis process during in vitro culture.

### Signal transduction

In this study, we found SGT1 homolog B (Bo3g045210) and SGT1 homolog A (Bo7g108890) in Zhonggan 628 under 32 °C vs. 25 °C and Zhonggan 628 under 32 °C vs. 87–534 under 32 °C, and their expression ratios were 2.0 and 2.2 and 1.58 and 1.76, respectively. In *Arabidopsis*, SGT1 contains SGT1a and SGT1b, and a double mutant could cause lethal embryos, suggesting that the SGT1a and SGT1b proteins are indispensable for plant development (Azevedo et al., 2006). In addition, SGT1 is also an indispensable resistance-related protein that has been shown to be a co-factor for HSP90 and HSP70. In *Arabidopsis*, the mutation of cochaperone SGT1b and HSP90 impairs responses to plant hormones, including auxin, gibberellic acid, and jasmonate (Zhang et al., 2015). In this study, the expression ratios of the two SGT1 proteins in the critical period of in vitro microspore development were found to be higher at 32 °C than at 25 °C. Moreover, SGT1 closely interacted with heat shock 70 kDa protein 5 (HSP70) (Fig. 6); although there has been no report describing SGT in traditional models such as barley *(Hordeum vulgare* L.), rapeseed (*B. napus* L.), tobacco (*Nicotiana* spp.), and wheat (*Triticum aestivum* L.) in microspore embryogenesis, there has been some reports about SGT1 in *Arabidopsis* in somatic embryogenesis, and we deduced that SGT1 may participate in early microspore embryogenesis by signal transduction.

### Cutin, suberine and wax biosynthesis

Cutin and waxes serve several functions, such as energy storage and protection of exposed surfaces from desiccation (Lardizabal et al., 2000). In this study, fatty acyl-CoA reductase (Bo1g139700, FAR) was involved in cutin, suberine and wax biosynthesis. In Zhonggan 628 under 32 °C vs. 25 °C for 24 h and Zhonggan 628 under 32 °C vs. 87–534 under 32 °C for 24 h, the expression ratios were 3.12 and 4.33, respectively. Although there have been no report about FAR in traditional models of microspore embryogenesis, we could speculate that FAR may be involved in embryo induction.

### Proposed model of microspore embryogenesis induced by heat shock in cabbage

**Figure 10:**
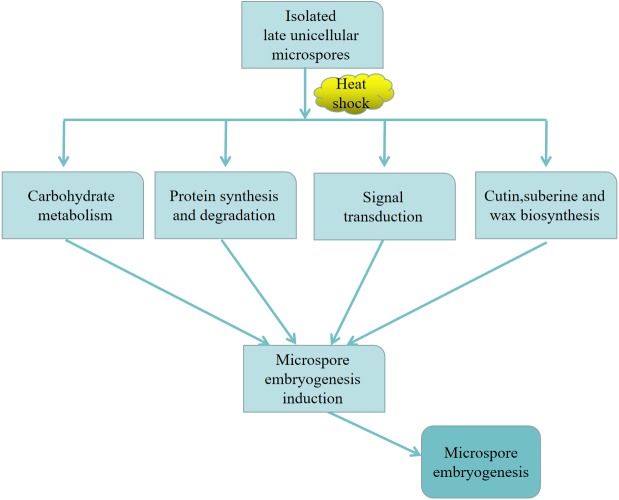
Proposed model of microspore embryogenesis induced by heat shock in cabbage.

Based on the above discussion, we proposed a model that might help to explain the regulatory networks governing microspore embryogenesis in cabbage (Fig. 10). First, in the protein synthesis and degradation process, the CDC48 and heat shock proteins (HSPs) in the isolated microspores responded to heat shock treatment. Then, CDC48 removed toxic waste from the ER, and HSPs were associated with the folding and unfolding of other proteins, as CDC48 and HSPs expression was higher at 32 °C than at 25 °C. At the same time, in the signal transduction process, SGT1, a cofactor of HSP70, stabilized microspore cellular signal transduction. Additionally, in cutin, suberine, and wax biosynthesis, FAR had higher expression at 32 °C, suggesting a potential role in microspore embryogenesis. Moreover, carbohydrate metabolism was involved in all these pathways by energy supply. Finally, the above mentioned regulatory interactions might lead to microspore embryogenesis. This model may help us to explore the mechanism of microspore embryogenesis induced by heatshock treatment. In the future, more functional analyses are needed to clarify this complex morphogenesis pathway.

## Conclusions

We investigated the molecular mechanisms of embryogenesis in vitro culture by proteomic profiles obtained by a label-free method in Zhonggan 628 and 87–534 after heat-shock treatment. 97 DEPs specifically identified only in Zhonggan 628 were primarily related to energy metabolism, protein synthesis and degradation, activation of the signal transduction pathway, and cutin, suberine and wax biosynthesis. SGT1 homolog B and A (SGT1), heat shock 70 kDa protein 5 (HSP70), cell division control protein 48 homolog A (CDC48), and fatty acyl-CoA reductase (FAR), identified based on protein-protein interaction and pathway enrichment analyses, might play important roles in cabbage microspore embryogenesis. This information will broaden our understandings of the molecular regulation of microspore embryogenesis induced by high-temperature treatment.

##  Supplemental Information

10.7717/peerj.8897/supp-1 Table S1 The primer sequences of 9 key proteins used for qRT-PCR

10.7717/peerj.8897/supp-2 Table S2 Differentially expressed proteins in all treatment groups

10.7717/peerj.8897/supp-3 Table S3 GO and KEGG analysis between 97 key DEPs

10.7717/peerj.8897/supp-4 Table S4 Protein-protein interaction analysis between DEPs

10.7717/peerj.8897/supp-5 Table S5 Carbohydrate metabolism and protein synthesis and degradation of key proteins

## Abbreviations

DEPs: differentially expressed proteins
D H: doubled haploid
LC-MS: liquid chromatography-tandem mass spectrometry
qRT-PCR: quantitative real-time-polymerase chain reaction
E R: endoplasmic reticulum
sHSP: small heat shock proteins
TCA: tricarboxylic acid cycle

## Additional Information and Declarations

### Data Availability

The following information was supplied regarding data availability: The raw measurements are available in the Supplemental Files.
